# Protocol for a multi-centered, stepped wedge, cluster randomized controlled trial of the de-adoption of oral chlorhexidine prophylaxis and implementation of an oral care bundle for mechanically ventilated critically ill patients: the CHORAL study

**DOI:** 10.1186/s13063-019-3673-0

**Published:** 2019-10-24

**Authors:** Craig M. Dale, Louise Rose, Sarah Carbone, Orla M. Smith, Lisa Burry, Eddy Fan, Andre Carlos Kajdacsy-Balla Amaral, Victoria A. McCredie, Ruxandra Pinto, Carlos R. Quiñonez, Susan Sutherland, Damon C. Scales, Brian H. Cuthbertson

**Affiliations:** 1 Lawrence S. Bloomberg Faculty of Nursing, University of Toronto, Toronto, Canada; 2 Trauma, Emergency and Critical Care, Sunnybrook Health Sciences Centre, Toronto, Canada; 3 Sunnybrook Research Institute, Toronto, Canada; 4 Department of Critical Care Medicine, Sunnybrook Health Sciences Centre, Toronto, Canada; 5 Florence Nightingale Faculty of Nursing, Midwifery and Palliative Care, King’s College, London, UK; 6 Department of Critical Care, St. Michael’s Hospital, Toronto, Canada; 7 Li Ka Shing Knowledge Institute, Toronto, Canada; 8 Department of Pharmacy, Mount Sinai Hospital, Toronto, Canada; 9 Leslie Dan Faculty of Pharmacy, University of Toronto, Toronto, Canada; 10 Interdepartmental Division of Critical Care Medicine, University of Toronto, Toronto, Canada; 11 Department of Medicine, University Health Network, Toronto, Canada; 12 Faculty of Dentistry, University of Toronto, Toronto, Canada; 13 Department of Dentistry, Sunnybrook Health Sciences Centre, Toronto, Canada

**Keywords:** Chlorhexidine, Critical care, Ventilator-associated pneumonia, Oral care, Stepped-wedge cluster randomized controlled trial, Process evaluation, Mechanical ventilation

## Abstract

**Background:**

Routine application of chlorhexidine oral rinse is recommended to reduce risk of ventilator-associated pneumonia (VAP) in mechanically ventilated patients. Recent reappraisal of the evidence from two meta-analyses suggests chlorhexidine may cause excess mortality in non-cardiac surgery patients and does not reduce VAP. Mechanisms for possible excess mortality are unclear. The CHORAL study will evaluate the impact of de-adopting chlorhexidine and implementing an oral care bundle (excluding chlorhexidine) on mortality, infection-related ventilator-associated complications (IVACs), and oral health status.

**Methods:**

The CHORAL study is a stepped wedge, cluster randomized controlled trial in six academic intensive care units (ICUs) in Toronto, Canada. Clusters (ICU) will be randomly allocated to six sequential steps over a 14-month period to de-adopt oral chlorhexidine and implement a standardized oral care bundle (oral assessment, tooth brushing, moisturization, and secretion removal). On study commencement, all clusters begin with a control period in which the standard of care is oral chlorhexidine. Clusters then begin crossover from control to intervention every 2 months according to the randomization schedule. Participants include all mechanically ventilated adults eligible to receive the standardized oral care bundle. The primary outcome is ICU mortality; secondary outcomes are IVACs and oral health status. We will determine demographics, antibiotic usage, mortality, and IVAC rates from a validated local ICU clinical registry. With six clusters and 50 ventilated patients on average each month per cluster, we estimate that 4200 patients provide 80% power after accounting for intracluster correlation to detect an absolute reduction in mortality of 5.5%. We will analyze our primary outcome of mortality using a generalized linear mixed model adjusting for time to account for secular trends. We will conduct a process evaluation to determine intervention fidelity and to inform interpretation of the trial results.

**Discussion:**

The CHORAL study will inform understanding of the effectiveness of de-adoption of oral chlorhexidine and implementation of a standardized oral care bundle for decreasing ICU mortality and IVAC rates while improving oral health status. Our process evaluation will inform clinicians and decision makers about intervention delivery to support future de-adoption if justified by trial results.

**Trial registration:**

ClinicalTrials.gov, NCT03382730. Registered on December 26, 2017.

**Electronic supplementary material:**

The online version of this article (10.1186/s13063-019-3673-0) contains supplementary material, which is available to authorized users.

## Background

Ventilator-associated pneumonia (VAP) was previously considered to have high morbidity and mortality and was the target for prevention strategies [1]. Evidence-based guidelines encouraged ICUs to adopt “ventilator bundles”, including the application of chlorhexidine gluconate mouth rinse, to prevent VAP [2–4]. However, two updated meta-analyses suggest chlorhexidine may cause excess mortality in some critically ill patients whilst failing to prevent VAP [5, 6]. Moreover, recent analyses demonstrate that the attributable mortality for VAP is low (1%) [7]. In combination, these data have placed guidelines for the use of oral care with chlorhexidine into question.

A variety of mechanisms have been proposed for the lack of effect of chlorhexidine oral rinse on VAP [8–11]. However, the biological mechanism for chlorhexidine causing excess mortality is less clear. Chlorhexidine could be directly toxic or it may trigger hypersensitivity reactions contributing to erosive mucosal lesions, predisposing patients to infection and respiratory failure [12, 13]. The additional burden of such complications in critically ill patients could negatively impact mortality.

Safe and effective oral care plays a critical role in the oral and systemic health outcomes of millions of people admitted to intensive care units (ICUs) worldwide each year for invasive mechanical ventilation [14]. Inadequate salivary flow associated with critical illness and oral intubation cause oral health dysfunction, including abnormal oropharyngeal colonization with bacteria [15, 16]. Aspiration of this abnormal oral bacteria leads to infection-related ventilator-associated complications (IVACs), which ultimately increase the duration of mechanical ventilation and costs of treatment [17]. Furthermore, resultant xerostomia is associated with severe pain and discomfort [18, 19].

With evidence for a lack of benefit in VAP prevention, as well as possible harm, immediate discontinuation of oral chlorhexidine in the ICU is a possible solution. However, the lack of a clear mechanism for harm, low to moderate quality of the evidence contributing to recent meta-analyses, and the potential for unintended consequences of rapid and widespread de-adoption all necessitate evaluation through scientifically rigorous de-adoption [20]. Therefore, we will conduct a multi-centered, stepped wedge, cluster randomized controlled trial (SW-CRT) of the de-adoption of oral chlorhexidine and implementation of a standardized oral care bundle in critically ill patients undergoing mechanical ventilation with an embedded process evaluation.

### Research questions

1. What are the effects of the de-adoption of oral chlorhexidine and introduction of a standardized oral care bundle on mortality in mechanically ventilated critically ill adults?
2. What are the effects of the de-adoption of oral chlorhexidine and introduction of a standardized oral care bundle on IVAC rates and oral health dysfunction scores in mechanically ventilated critically ill adults?

## Methods/design

### Design

**Fig. 1:**
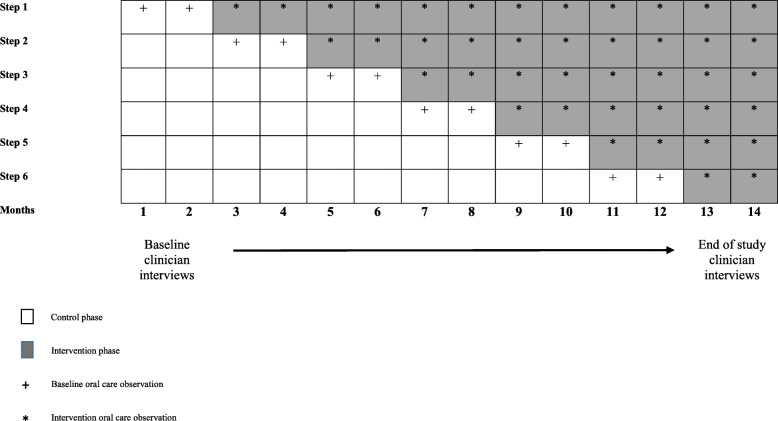
Stepped-wedge schedule of the CHORAL study. Clusters sequentially cross over from control phase to intervention phase. Observational data collection is depicted during control (*plus sign*) and intervention (*asterisks*) periods

The CHORAL study is a SW-CRT. Each cluster (defined as a single ICU) will be randomly allocated to receive the intervention according to a staggered implementation schedule in one of six sequential steps comprising 2-month intervals. There will be six clusters in total with all clusters commencing with a 2-month control period (baseline) in which standard of care includes chlorhexidine oral care for IVAC prevention. Each cluster will maintain chlorhexidine oral care until they are allocated to crossover from control to intervention according to the randomization schedule. The study completes with a 2-month period during which all clusters have fully de-adopted chlorhexidine and implemented the standardized oral care bundle. Total study duration will be 14 months (Fig. 1). We follow CONSORT cluster trial extension guidelines for SW-RCTs in the design of this study [21]. The SPIRIT checklist is provided in Additional file 2.

The key rationale for our selection of a SW-CRT design includes: (1) avoidance of contamination of the intervention for control participants that is a risk with individual patient randomization; (2) provision of the intervention education at the cluster level; (3) intention to leave the oral care intervention in place at study end; and (4) measurement of outcomes at the level of both clusters and individual patients.

A mixed-methods process evaluation will be conducted as an adjunct to the main trial. Guided by a framework for process evaluation of complex interventions in cluster randomized trials, we will study the processes supporting implementation of our intervention. The objective of our process evaluation is to provide a clear description of implementation processes and to facilitate understanding of the main trial results [22].

### Study setting and participants

The CHORAL study will be conducted in six adult ICUs in five university-affiliated hospitals in Toronto, Canada.

### Inclusion and exclusion criteria

#### Unit inclusion for SW-CRT

We approached units meeting the following criteria for participation: (1) adult ICU capable of providing invasive mechanical ventilation; (2) existing intention to de-adopt oral chlorhexidine prophylaxis; (3) contribution of patient demographic, treatment, and outcome data to the Toronto Intensive Care Observational Registry (iCORE); and (4) admission of at least 50 ventilated patients per month. Exclusion criteria: units not meeting inclusion criteria.

#### Patient inclusion criteria for SW-CRT

All adult patients who receive invasive mechanical ventilation in the participating ICUs and are able to receive standardized oral care can be included. For mortality and IVAC rates we will exclude those patients who start in the control group and cross-over to the intervention group.

### Randomization and allocation concealment

Randomization of ICUs to the six sequential steps will be computer-generated by the study statistician. Each ICU will be randomly allocated to start the study intervention according to the randomized staggered implementation schedule and the sequence will be concealed until interventions are assigned. Clusters agreed to participate with the understanding that order of allocation to the intervention would be unknown in advance of randomization. Within control and intervention periods, random dates of observation at each site will be computer generated to collect oral health dysfunction scores and establish intervention fidelity in a subset of patients.

### Blinding

This is a non-blinded study. Clinical and study staff cannot be blinded to study allocation due to the nature of the intervention. Clinical staff in all clusters will be provided with initial education regarding the study intervention and ongoing education to reinforce delivery of the standardized oral care bundle. Independent iCORE assessors of mortality and IVAC outcome data will be blinded.

### Pre-intervention phase

#### Oral care observations

In the 2 months preceding de-adoption of chlorhexidine and introduction of a standardized oral care bundle at each site, a trained standardized assessor will use structured observational tools to document baseline oral care delivery, including chlorhexidine concentration and application method [23]. We will document pain presence (yes/no) during oral care using the Critical-Care Pain Observation Tool (CPOT; range 0–8, score > 2 indicating pain) [24], and oral health dysfunction using the Beck Oral Assessment Scale (BOAS) [25] on randomly selected dates. The CPOT was recently validated for detecting pain presence in non-verbal ICU patients during oral care delivery and the BOAS tool has been previously validated in the ICU population [26]. For patients able to self-report (mouth words, nod, point), we will also measure symptom intensity using a 0–10 intensity-rating scale for (i) oropharyngeal dryness and (ii) procedural oral pain [27] immediately following the oral care delivery. We aim to observe a minimum of 25 patients in each ICU during this baseline phase (150 total).

### Intervention phase

Four weeks prior to the intervention phase, an investigator or designate will deliver intensive education targeting clinician knowledge, attitudes, and behaviors regarding the new oral care intervention to ICU cluster leads using a train-the-trainer model [28]. Cluster leads will comprise an ICU physician-nurse (or physician-pharmacist) dyad supported by unit-level oral care nurse champions. We will utilize an integrated knowledge translation (iKT) approach to facilitate practice change, including an iKT oral care tool kit supporting our oral care intervention [29] comprising a detailed oral care protocol (Table 1), touchpoint video (an edited video of ICU patient experiences and recommendations for oral care), and expert video instruction on oral care delivery (Additional file 1). To minimize bias, cluster leads will distribute the iKT oral care tool kit to frontline ICU nursing and allied health staff 2 weeks prior to the intervention phase.

**Table 1:** Oral care bundle

Comprehensive oral care Q12 hours	Equipment	Procedure
1. Oral assessment	•Flashlight •Tongue depressor •Gloves •Face shield	•Explain procedure to patient •Gently open mouth or use mouth prop •Inquire about mouth/throat pain (0–10 NRS) •Use CPOT tool to evaluate pain in non-verbal pt. •Treat pain prior to proceeding
2. Tooth brushing	•Yankauer •12 or 14 French flexible catheter •Small soft-bristle or suction toothbrush •Sponge swabs •Sterile water •Gloves •Face shield	•Explain procedure to patient •Perform hand hygiene •Elevate HOB 30–45 degrees as tolerated •Use oral prop to open mouth as needed •Oral suction with Yankauer or sterile flexible catheter to remove secretions that may migrate down airway •Moisten toothbrush with sterile water •Connect suction toothbrush to continuous suction if applicable •Brush accessible teeth and gums for 2 full minutes or 30 s per quadrant; brush in one continuous line LUQ > RUQ > RLQ > LLQ •Gently brush tongue
3. Mouth and lip moisturizer	•Swabs •Mouth moisturizer/saliva replacement or sterile water •Gloves •Face shield	•Explain procedure to patient •Use oral prop to open mouth as needed •Use 1–3 swabs to apply moisturizer to oral mucosa, tongue, and lips
4. Deep oral suctioning	•Yankauer or flexible catheter •Gloves •Face shield	•Explain procedure to patient •Use oral prop to open mouth as needed •Deep oropharyngeal suction (above the cuff) to remove pooled secretions
Maintenance oral care Q4 hours and PRN	Equipment	Procedure
Mouth and lip moisturizer	•As above	•As above
Oral secretion removal	•As above	•As above

*CPOT* Critical-Care Pain Observational Tool, *HOB* head of bed, *PRN* pro re nata or “as needed”

The following oral care interventions will commence in the intervention phase for each step and become standard of care:

1. Intervention 1 (oral chlorhexidine de-adoption): Each cluster will de-adopt oral chlorhexidine from routine practice for all invasively ventilated patients. Use of routine oral chlorhexidine will not be permitted during the intervention period with the exception of patients prescribed chlorhexidine for non-standard care.
2. Intervention 2 (oral care protocol): This two-part, nurse-led intervention incorporates comprehensive and maintenance oral care activities. Comprehensive care involves twice daily (morning and evening) a) oral assessment (inspection of the oral space, teeth and gums); b) tooth brushing; c) oral/lip moisturization; and d) suctioning oropharyngeal secretions above the cuff. Maintenance care involves, at a minimum of every 4 h and as needed, a) oral/lip moisturization; and b) suctioning oropharyngeal secretions above the cuff (Table 1).

One month after allocation to the intervention, a trained assessor will begin to observe fidelity of delivery of the oral care protocol and assess patient oral health status using the BOAS, CPOT, and patient self-report of oral health symptom intensity on randomly selected dates. We will provide each cluster lead with written feedback on these observations for dissemination to frontline ICU staff. For sites identified as having low intervention fidelity during audits (< 70% compliance across all bundle components), we will provide additional education and intensified audit frequency. We estimate observing a minimum of 25 patients in each ICU during the intervention phase (150 total).

### Outcome measures

The primary outcome is ICU mortality measured at the level of the patient. Secondary outcomes include: (1) IVAC rates; (2) and oral care dysfunction (BOAS) scores measured at the patient level.

### Data collection

iCORE is a local clinical registry that collects de-identified patient demographic and clinical data, including severity of illness, antibiotic usage, IVAC rates, and patient outcomes. We will use the iCORE registry to collect patient demographic and treatment characteristics (i.e., age, sex, co-morbidity, admission diagnosis, nature of admission (elective or emergent), and severity of illness (APACHE III score), as well as to determine outcome data including IVAC rates and ICU mortality. The BOAS tool [25], oral care delivery compliance, pain, and other oral symptoms will be prospectively measured by the investigator team.

### Sample size

#### Mortality

Recent systematic reviews [30] show that chlorhexidine increased mortality with an odds ratio (OR) of 1.25 (95% confidence interval (CI) 1.05–1.50). This corresponds to a risk ratio of 1.18 (95% CI 1.04–1.33) and an absolute reduction in mortality of 5% (95% CI 1%–6%). Six clusters averaging 50 ventilated patients per month for 14 months for a total of 4200 patients, assuming an intracluster correlation (ICC) of 0.001, provides about 80% power to detect an absolute reduction in mortality of 5.5% from a baseline rate of 26% [6, 31, 32].

#### Oral health dysfunction

The BOAS scale ranges from 5 to 20, with a score of 5 equivalent to no oral problems and 20 equivalent to the worst possible oral health state. Previous ICU studies report a mean (standard deviation) score of 10 (2) [26]. Taking into account clustering (DEF = 1 + (n − 1) × ICC with an ICC = 0.05 to 0.2), the design effect would range from 3.5 to 10 and therefore the sample size ranges from 86 to 300. We will observe a minimum of 25 patients per ICU at control and intervention phases. With 150 patients in each time period we have 80% power to detect a two-point decrease in the BOAS in the intervention group.

### Analysis and evaluation

#### Statistical analysis

We will describe our patient cohort and treatment characteristics using means and standard deviations or medians and interquartile ranges dependent on data distribution for continuous variables and frequencies and percentages for categorical variables. To account for clustering of patients within sites, the primary outcome, mortality, will be analyzed using a generalized linear mixed model (with logit link and binary distribution) adjusting for time to account for secular trends as well as important prognostic factors such as age, sex, APACHE III score, and comorbidities. We will inspect missing data and if less than 5% are missing we will use imputation by the median values for the continuous variables and mode for the categorical ones. If more than 5% of the sample has missing values we will use multiple imputations. Differences between the control and intervention periods and time trends will be estimated using OR and their 95% CIs. IVAC counts with offset number of ventilator days per patient will be analyzed using a generalized linear mixed model with log link and Poisson or negative binomial distribution as dictated by the distribution of the data. For mortality and IVAC rates we will exclude those patients who start in the control group and cross-over to the intervention group. BOAS will be summarized using mean and standard deviation and analyzed using linear mixed models (adjusting for clustering of patients within sites) with treatment phase as an independent variable. We will report the CPOT (oral pain during care delivery) scores, and patient self-report (pain, dryness) outcomes, as medians and IQR and analyze using the Wilcoxon rank sum test to compare the pre-intervention and intervention groups.

### Process evaluation

Our embedded mixed methods process evaluation will examine the reach of oral care intervention education, stakeholder response to chlorhexidine de-adoption and implementation of a standardized oral care bundle, and fidelity of oral care processes [22].

#### Pre-and post-intervention oral care fidelity

In the 2 months preceding chlorhexidine de-adoption and implementation of a standardized oral care bundle, and then each month following implementation, trained study team members will collect oral care fidelity data using a structured observational tool on randomized dates. We will observe a minimum of 25 patients per ICU at pre-intervention and intervention phases for a total of 150 patients in each time period. We will report oral care fidelity as medians and IQR and analyze using the Wilcoxon rank sum test to compare the pre-intervention and intervention groups.

#### Pre-and post-intervention qualitative interviews

During pre-intervention and end-of-study periods, we will recruit and interview eligible (i.e., working in a participating unit for ≥ 3 months) ICU nurses, respiratory therapists, pharmacists, physiotherapists, speech-language pathologists, and physicians. Pre-intervention interviews will target current oral care practices in addition to anticipated barriers and facilitators to the de-adoption of chlorhexidine and implementation of a standardized oral care bundle. End-of-study interviews will address the reach of the educational delivery, stakeholder response to the intervention, perceptions of oral care bundle fidelity, and contextual factors (e.g., secular trends) impacting the study. Sixty clinician interviews (ten per site) in each time period will occur by telephone or in person at the hospital, based on participant preference. We will use qualitative content analysis (CA), a method for describing the content of communication in an objective and systematic manner for transcribed clinician interviews [33]. Two investigators will independently code interviews to enhance rigor.

## Discussion

To our knowledge, the CHORAL study is the first SW-CRT designed to evaluate the effect of de-adopting chlorhexidine oral rinse and implementation of a standardized oral care bundle on ICU mortality, IVACs, and oral health status. In response to multiple independent meta-analyses suggesting the possibility of harm, we aim to conduct a large prospective trial with sufficient statistical power to detect mortality differences between patients receiving chlorhexidine oral care and those receiving non-chlorhexidine oral care. The prospective nature of the trial is important as the mortality signal of concern is only present in meta-analyses [10, 11].

Clear evidence regarding the effect of oral chlorhexidine de-adoption on our primary outcome of mortality will clarify concerns of preventable harm. Secondary outcomes, including IVAC rates and oral health status following the de-adoption of oral chlorhexidine and delivery of a standardized oral care bundle, will enhance knowledge regarding the impact of our intervention on patient-centered outcomes, including infection and oral health dysfunction. As chlorhexidine is presently identified as an indicator of high-quality ICU care [6], our study results will prompt re-examination of professional society guidelines.

We have chosen a SW-CRT design due to the expressed desire of participating clusters to de-adopt chlorhexidine and the requirement to deliver the intervention and measure outcomes at the level of individual patients and at the level of the cluster. The CHORAL study introduces a substantial change in longstanding oral care practices which requires provision of education to large groups of care providers across discrete units. An important advantage of the study design is the ability to deliver education in smaller groups (clusters) at pre-defined time-points. This approach will avoid contamination between intervention and control groups seen in parallel randomized controlled trial (RCT) design and alleviate ethical concerns about withholding or blinding of interventions in RCTs.

As effective methods to de-adopt established ICU practices are unclear, our process evaluation will provide clinicians and policy makers with vital information about how the intervention was actively delivered [18]. This is important as ambiguity regarding the composition and delivery of ICU processes threatens interpretation, replication, and research investment. In addition, our embedded process evaluation will clarify helpful strategies for effective education, thereby supporting future integration of oral care interventions across varying practice contexts [29].

## Trial status

Protocol version 14/Aug/2017 commenced on January 31, 2018 and will proceed until March 31, 2019.

## Additional files

Additional file 1: CHORAL educational videos. (DOCX 13 kb)

Additional file 2: SPIRIT 2013 Checklist: Recommended items to address in a clinical trial protocol and related documents*. (DOCX 39 kb)

## Data Availability

The datasets generated during the course of this study may be made available through the corresponding author upon reasonable request.

## Abbreviations

BOAS: Beck Oral Assessment Scale
CA: Content analysis
CDC: Centers for Disease Control
CI: Confidence interval
CPOT: Critical-Care Pain Observation Tool
CTO: Clinical Trials Ontario
iCORE: Intensive Care Observational Registry
ICU: Intensive care unit
iKT: Integrated knowledge translation
IQR: Interquartile range
IVAC: Infection-related ventilator-associated complications
SW-CRT: Stepped-wedge cluster randomized controlled trial
VAP: Ventilator-associated pneumonia
