# Supplementary material for: Protocol for a multi-centered, stepped wedge, cluster randomized controlled trial of the de-adoption of oral chlorhexidine prophylaxis and implementation of an oral care bundle for mechanically ventilated critically ill patients: the CHORAL study
Source: Trials. 2019 Oct 24;20:603. doi: 10.1186/s13063-019-3673-0 (PMC6814100; doi:10.1186/s13063-019-3673-0)
Supplement: Supplementary file 1 — CHORAL educational videos. (DOCX 13 kb) [file 13063_2019_3673_MOESM1_ESM.docx]

Additional file 1

1. Comprehensive/Maintenance Oral Care instructions <https://youtu.be/8Uh42xv1bAE>

2. Patient/family experiences and recommendations: <https://youtu.be/vAUDeZ9L6s8>
